# Nurses’ Workplace Bullying Experiences, Responses, and Ways of Coping

**DOI:** 10.3390/ijerph17197052

**Published:** 2020-09-26

**Authors:** Sun Yee Yoo, Hye Young Ahn

**Affiliations:** 1 Department of Nursing, Gwangju University, Gwangju 61743, Korea; syyoo@gwangju.ac.kr; 2 College of Nursing, Eulji University, Daejeon 34824, Korea

**Keywords:** workplace bullying experiences, workplace bullying response, ways of coping, bullying, nurse

## Abstract

Workplace bullying is a serious problem that hinders the provision of quality care services by seriously affecting their physical, psychological, and social health status. Workplace bullying experiences refer to verbal and nonverbal harassment, work-related harassment, and external threats. Workplace bullying responses are negative reactions that occur in individuals who have experienced workplace bullying, while coping is the process by which an individual copes with stress. This study aims to analyze the relationship between nurses’ workplace bullying experiences, responses, and ways of coping. We studied 113 nurses working in hospitals, analyzed the data using SPSS 25.0. We found that the more positive use of the positive viewpoint, the lower the bullying reaction in the workplace (r = −0.268, *p* = 0.004). Workplace bullying responses were lower as more positive ways of coping were used (r = −0. 268, *p* = 0.004). In conclusion, nurses who experience bullying in the workplace should be supported by the hospital organization and within the nursing organization, and a receptive nursing culture should be established. There is also a need for an intervention plan that allows nurses to use positive ways of coping with workplace bullying experiences.

## 1. Introduction

Among other types of violence at the workplace, bullying is an act of individuals or groups harming a certain individual by constantly trying to exclude him/her from a social context [1]. In Korea, in the 2000s, harassment of nurses in the workplace became an issue, and related research began in earnest. The social demand for prevention of bullying in the workplace, including the culture of “Taewoom,” a word symbolizing bullying in the Korean nursing community, has increased. Therefore, from July 16th, 2019, the “Workplace Bullying Prohibition Act” was enforced, which prohibits employers or workers from using their position or relationship advantage at work to cause physical or mental pain to other workers [2]. Currently, Korean society is reforming the wrong nursing culture within nursing organizations through the enforcement of this law. 

Workplace bullying experiences among nurses is defined as a negative act in a working situation characterized by a power imbalance, which can exert serious effects on the organization, as well as on individual nurses [3]. In hospitals, there are many types of bullying offenders, including patients, caregivers, doctors, and nurses. Of these, bullying most frequently occurs among nurses and is particularly committed by a colleague rather than by a manager [4].

Nursing, which is related to human life, is unpredictable, intensive work, and involves sundry emergencies. For this reason, nurses may work in a stressful situation and may be more likely to be bullied [5]. Nurses bullied at a workplace try to accept it instead of demanding the situation improve [6]. Since offenders are principally those in higher positions, such as senior nurses or managers, nurses in lower positions in the Korean society, based on the Confucian idea, tend to cope with the situation by bearing or evading it [7]. They cope with the situation passively by enduring and neglecting it and by regarding the attempt to face negative acts during work hours as a “part of nursing” [8]. 

Nurses bullied at the workplace may complain of mental symptoms, such as depression, anxiety, and fear, as well as physical symptoms, such as fatigue, headaches, and palpitations [9]. They are also more susceptible to burnout and turnover intention and have lower levels of organizational commitment and nursing productivity. This demonstrates that workplace bullying experiences can actually affect nurses’ job attitudes and organizational performance [8]. These negative personal and organizational effects of workplace bullying experiences on nurses can result in socio-economic loss.

Individuals faced with a crisis like bullying attempt to find a solution to the problem, and such an attempt is called coping [10]. A literature review regarding nurses’ experience of being bullied at the workplace shows that nurses bullied at the workplace can face the reality of being bullied and grow as nurses through several trials and errors, as well as through the formation of relationships with their colleagues; however, if they fail to get over the shock of being bullied, to make an unsuccessful effort to solve the problem, or unsuccessfully bear the frustrating result, they may give up their job altogether [5]. 

Problem-focused coping is the most desirable and effective coping ability. It involves thoughts and actions that accurately analyze the cause of a problem in a stressful situation and plan to actively solve it [11]. Based on this, the established hypothesis for this study was that there is a negative correlation between problem-focused coping, workplace bullying experiences, and workplace bullying response. Hypothesis verification can provide information on how to deal with nurses who should be first trained to solve problems related to bullying in the workplace. Moreover, to solve the workplace bullying experiences, the individual’s specific ways of coping may control bullying responses and contribute to individual nurses’ growth. It is necessary to analyze workplace bullying experiences, responses, and ways of coping. 

However, little research has been conducted on the association between the ways of coping with bullying and bullying responses in nursing practice. This study aimed to examine nurses’ experiences of workplace bullying, responses, and ways of coping; analyze the association among these factors; and provide basic data that could help control the negative effects of workplace bullying experiences and develop effective coping strategies.

The purpose of this study was to examine nurses’ workplace bullying experiences, workplace bullying responses, and ways of coping; analyze the correlation among these factors; provide basic data necessary to reduce the negative effects of workplace bullying experiences; and develop strategies to improve the quality of nursing. Its specific objectives were as follows:

- To examine the participants’ workplace bullying experiences, workplace bullying responses, and ways of coping;
- To determine ways of coping based on the participants’ general characteristics;
- To determine the correlation among the participants’ workplace bullying experiences, workplace bullying responses, and ways of coping.

## 2. Materials and Methods 

### 2.1. Research Design

This study involved descriptive research aimed at examining nurses’ workplace bullying experiences, workplace bullying responses, and ways of coping and at determining correlation among these factors. 

### 2.2. Research Participants

This study targeted nurses with more than six months of clinical experience working at university hospitals in D city and C province. According to the definition [12] of workplace bullying experiences as repetitive bullying that lasts for more than six months, nurses that had been employed for less than six months were excluded. The number of subjects for the correlation analysis between workplace bullying experiences, workplace bullying response, and response methods of nurses was based on previous studies [13]. When the significance level of 0.05, the effect size of 0.02, and power of 0.8 predictor was 11, the minimum sample size using the GPower sample size estimator v.3.1.9.2. program (Universität Kiel, Kiel, Germany) was 95. The questionnaire was distributed to a total of 120 people in consideration of the 20% dropout rate, but (due to the lack of content in the responses), seven inappropriate questionnaires were excluded. A total of 113 questionnaires were used for data analysis.

### 2.3. Research Instrument

#### 2.3.1. Workplace Bullying Experiences

Workplace bullying experiences refer to verbal and nonverbal harassment, work-related harassment, and external threats that consistently appear between individuals and other individuals or within individuals and groups amid an inefficient organizational culture and power imbalance [14]. 

The Workplace Bullying in Nursing-Type Inventory (WPBN-TI) developed by Lee et al. was used to measure workplace bullying experiences [7]. Each item was rated on a four-point Likert scale—1 for totally disagree, 2 for somewhat disagree, 3 for somewhat agree, and 4 for totally agree—with a higher score meaning greater exposure to workplace bullying experiences. At the time of its development, the tool was composed of 10 items concerning verbal abuse and alienation, four concerning inappropriate task assignment, and two concerning physical threat. The original tool had a Cronbach’s α of 0.91. In this study, it had a Cronbach’s α of 0.917 for reliability. 

#### 2.3.2. Workplace Bullying Response

The bullying reaction in the workplace refers to a phenomenon that increases negative energy in the organization, along with physical and psychological withdrawal in individuals who experience workplace bullying [14]. 

The Workplace Bullying in Nursing-Consequence Inventory (WPBN-CI) developed by Lee was used to measure workplace bullying responses [9]. Each item was rated on a four-point Likert scale—1 for totally disagree, 2 for somewhat disagree, 3 for somewhat agree, and 4 for totally agree—with a higher score meaning a stronger impact of workplace bullying experiences. At the time of its development, the tool was composed of eight items concerning physical and psychological withdrawal, two concerning a lower quality of nursing, and three concerning increased distrust with a Cronbach’s α of 0.90. In this study, it had a Cronbach’s α of 0.847 for reliability.

#### 2.3.3. Way of Coping

Coping is an active process performed by individuals to minimize damage, and respond to crises, freeing them from internal and external stress factors [10]. The scale developed by Lazarus and Folkman [10], and translated and revised by Han [15], was used to measure ways of coping.

It was composed of 33 items: Eight in the problem-focused domain, five in wishful thinking, six in detachment, seven in seeking social support, four in focusing on the positive, and three in tension reduction. Each item was rated on a four-point Likert scale—from 1 for totally disagree to 4 for totally disagree—with a higher score meaning a higher likelihood of using a way of coping in each domain. Cronbach’s α was 0.790 in the study of Han et al. and 0.818 in this study.

### 2.4. Data Collection

The data collection period for this study was from August 1st to September 30th, 2019. For data collection, the chief of the nursing department in the institution was given an explanation of the purpose of the study and was asked to permit data collection. 

We visited each ward of the three university hospitals that allowed the survey, and explained to the nurses the purpose and method of the study. We guaranteed anonymity and autonomy for participation in the study, advised of the possible benefits and disadvantages, and the possibility of abandoning the survey. Subjects who wished to participate in the survey were asked to sign a research consent form to collect data, and a questionnaire was distributed to nurses who signed the consent to participate in the study. The questionnaire was provided in individual opaque envelopes with double-sided tape attached.

Immediately after the subject responded to the questionnaire, the questionnaire was placed in an opaque individual envelope, sealed with double-sided tape, and collected by the researcher. Immediately after the researcher personally collects the questionnaire, a predetermined gift was provided as a token of gratitude to the subjects who completed the survey.

### 2.5. Ethical Considerations

This study was approved by the institutional review board (EU19-55) of Eulji University. The questionnaire was distributed only to the subjects who agreed to participate in the study. The collected questionnaires were kept safely in the place where the locker was located. Participants were informed that the collected surveys would be kept for three years, after which they would be destroyed using a document shredder.

### 2.6. Data Analysis

The collected data were analyzed using the SPSS 25.0 program (IBM, Armonk, NY, USA). The general characteristics of the subject were gender, marital status, education, type of duty, age (years), religion, working unit, and the total length of career (years). Data were analyzed by frequency and percentage according to each general characteristic. 

Descriptive statistical methods of frequency, mean, and standard deviation were used to determine the subject’s workplace bullying experiences, response, and way of coping. The response method, according to the general characteristics of the subjects, was analyzed by t-test and ANOVA.

Finally, The correlation among the participants’ workplace bullying experiences and sub-factors (verbal attacks and alienation, improper work instructions, physical threats), workplace bullying response and sub-factors (physical and psychological withdrawal, poor quality of patient care, increasing distrust), and the six ways of coping (problem-focused, wishful thinking, detachment, seeking social support, focusing on the positive, tension reduction) was analyzed. The correlation between variables was analyzed using Pearson’s correlation coefficient.

## 3. Results

### 3.1. Participants’ General Characteristics

Of the 113 participants, 106 (93.8%) were female, and 7 (6.2%) were male. Ninety-two (81.4%) were unmarried, and 21 (18.6%) were married. Fifty-one (45.1%) were three-year nursing school graduates, 57 (50.4%) were four-year nursing school graduates, and 5 (4.4%) were graduate school graduates or at higher education levels.

One hundred and six (93.8%) were on shift work, and 7 (6.2%) were on full-time work. Eighty-one (71.7%) were in their twenties, 29 (25.7%) in their thirties, and 3 (2.7%) in their forties or older. Forty (35.4%) had a religion, and 73 (64.6%) had no religion.

Forty-five (39.8%) worked in the medicine ward, 21 (18.6%) in the surgery ward, 38 (33.6%) in special departments (e.g., intensive care units, operation rooms, and emergency rooms), and 30 (8.0%) in other departments (e.g., newborn unit, nursing department, artificial kidney unit, and physician assistant). Fifteen (13.3%) had nursing careers lasting less than 1 year, 54 (47.8%) had careers lasting 1 to <5 years, 31 (27.4%) had careers lasting 5 to <10 years, and 13 (11.5%) had careers lasting at least 10 years (Table 1).

### 3.2. Participants’ Workplace Bullying Experiences, Responses, and Ways of Coping

Participants scored an average of 29.39 (±7.33) out of 64 for workplace bullying experiences. As for its sub-factors, they scored an average of 18.58 (±5.08) out of 40 for verbal abuse and alienation, an average of 8.17 (±2.32) out of 16 for inappropriate task assignment, and an average of 2.64 (±0.92) out of 8 for physical threat. They scored an average of 30.98 (±6.09) out of 52 for workplace bullying responses. 

As for sub-factors, they scored an average of 19.75 (±4.10) out of 32 for physical and psychological withdrawals, an average of 4.57 (±1.26) out of 8 for lower quality of nursing, and an average of 6.66 (±1.99) out of 12 for increased distrust. As for their ways of coping, they scored an average of 22.19 (±2.51) out of 32, 12.6 (±1.76) out of 20 for wishful thinking, 13.99 (±2.14) out of 24 for detachment, 18.21 (±2.28) out of 28 for seeking social support, 10.59 (±1.74) out of 16 for focusing on the positive, and 7.42 (±1.57) out of 12 for tension reduction (Table 2).

### 3.3. Participants’ Workplace Bullying Experiences, Responses, and Ways of Coping by Their General Characteristics

As for variation in the participants’ ways of coping based on their general characteristics, wishful thinking showed statistically significant variation by gender (t= −2.31, *p =* 0.023) and religious status (t= −2.63, *p =* 0.010); that is, females scored higher for their way of coping based on wishful thinking than males. Religious participants scored higher for their way of coping based on wishful thinking compared to the non-religious.

Among the sub-areas of ways of coping, tension reduction showed statistically significant variation by marital status (t= −2.99, *p =* 0.003) and working type (t= −2.26, *p =* 0.026); that is, the unmarried scored higher for their way of coping based on tension reduction than the married; whereas, the participants on shift work scored higher for their way of coping based on tension reduction than those on full-time work (Table 3).

### 3.4. Correlation among Workplace Bullying Experiences, Responses, and Ways of Coping

Workplace bullying experiences were significantly positively correlated with workplace bullying responses (r = 0.52, *p <* 0.001) and was positively correlated with physical and psychological withdrawal (r = 0.29, *p =* 0.002), lower quality of nursing (r = 0.60, *p <* 0.001), and increased distrust (r = 0.59, *p <* 0.001) among its sub-factors.

Workplace bullying experiences were significantly negatively correlated with problem-focused ways of coping (r = −0.23, *p =* 0.013) and ways of coping from a focusing on the positive (r = −0.23, *p =* 0.016).

Workplace bullying response was significantly negatively correlated with the way of coping from a focusing on the positive (r = −0.27, *p =* 0.004), which was significantly negatively correlated with physical and psychological withdrawal (r = −0.20, *p =* 0.031), lower quality of nursing (r = −0.25, *p =* 0.008), and increased distrust (r = −0.24, *p =* 0.009) among its sub-factors.

Of the sub-factors for workplace bullying response, lower quality of nursing was significantly negatively correlated with problem-focused ways of coping (r = −0.187, *p =* 0.047), and increased distrust was significantly positively correlated with detachment (r = −0.234, *p =* 0.013) (Table 4).

## 4. Discussion 

This study aimed to examine nurses’ workplace bullying experiences, responses, and ways of coping and to determine the association among these factors. The nurse practitioners in this study complained of helplessness, depression, stress, insomnia, and physical discomfort after being bullied at the workplace. This result is consistent with the finding of a literature review that nurses exposed to horizontal violence may experience such psychological problems as distress, anger, fear, and anxiety, as well as some physical symptoms, such as headaches, sleep disorder, and indigestion [16]. In addition, in this study, it was found that nurses who experienced bullying in the workplace made a lot of mistakes in their work and had a desire to change jobs, similar to the results of previous studies [17]. This result demonstrates that the experience of being bullied at a workplace can have physically and mentally negative effects on individual nurses, lower the quality of the nursing service, and increase turnover intention. Therefore, hospital organizations need to give support to nurses with the aim of identifying individual nurses’ negative responses to the experience of being bullied at the workplace and coping with them. Nursing organizations need to establish a receptive nursing culture that permits nurses to reveal their experiences of being bullied at the workplace or resultant negative responses or distress and to create a site for communication among them.

Workplace bullying responses were negatively correlated with ways of coping from focusing on the positive; that is, the more likely nurses were to apply ways of coping from focusing on the positive, the less likely they were to show workplace bullying responses. Nurses who tried to see the positive side of an event and regarded the present issue as a driving force of growth were less likely to show negative responses to workplace bullying. This result is supported by the finding of a literature review that nurses experiencing the last phase of relief during the process of coping with workplace bullying accepted their seniors’ bullying as a kind of training for their own growth [18].

It is necessary to improve the systematic organizational culture and environment with the objective of solving the fundamental problems of workplace bullying [18,19]. However, since this takes a long time to change, and problem-solving needs to focus on the position of the bullied, priority should be given to the development of a plan to maintain well-being for individuals. On the basis of the finding of a literature review that education for positive coping with a problem situation could reduce the negative effects of being bullied [20], it is necessary to develop an intervention for nurses to apply ways of coping with workplace bullying from a positive perspective.

Among the sub-areas of workplace bullying responses, lower quality of nursing was negatively correlated with problem-based ways of coping; that is, nurses making a plan to analyze and settle a problem situation were less likely to make a mistake related to work or were less willing to resign, due to the offender after being bullied at the workplace. This result implies that while they regarded themselves as victims of bullying, they controlled the negative responses to bullying through their own strong belief and efforts to solve the problem, instead of avoiding the situation they faced. Many researchers have been worried about nurses’ strong turnover intention due to workplace bullying [8,21,22]. On the basis of these results, therefore, it is necessary to add education and training related to problem-based ways of coping with the plan for intervention to reduce turnover intention caused by workplace bullying.

Among the sub-areas of workplace bullying responses, increased distrust was positively correlated with detachment-based ways of coping; that is, nurses trying to endure in overcoming a problem situation, believing that time will solve, can have a negative idea of nursing, as well as a loss of belief in the hospital or the manager after being bullied at the workplace. Many nurses have tried to forget the problem caused by the experience of being bullied at the workplace and avoided it [8]. The failure to cope well with bullying responses can form distrust in the organization and a negative idea of nurses, which is deeply associated with the turnover intention [9,13]. Even those nurses forming a negative image of the hospital and nurses after being bullied at the workplace need to work; thus, they seem to try to neglect the present problem, and the repetition of this situation can cause them to stop their work. Therefore, managers in each unit of the hospital need to identify cases of bullying through an in-depth and steady interview with nurses and help them cope well with their situation.

## 5. Conclusions

This study involved descriptive research aimed at examining nurse practitioners’ workplace bullying experiences, responses, and ways of coping; analyzing the association among these factors; and providing the basic data necessary to develop a strategy for reducing negative results from workplace bullying experiences and improving the quality of nursing. Nurses applying ways of coping from a positive perspective were less likely to show workplace bullying responses. Those applying problem-based ways of coping were less likely to make a mistake related to work or were less willing to resign. Those applying neglect-based ways of coping were more likely to have distrust in the organization and a negative idea of nurses after being bullied at the workplace. It is, therefore, necessary to create an intervention program for nurses to cope with workplace bullying experiences from a positive perspective and give education and training to them so that they can analyze and settle the problem situation. Nurses in each unit of the hospital need to identify the cases of bullying and help victims cope well with the situation through in-depth interviews.

Based on these results, it can be seen that the consideration of individual coping methods should be included when planning an interventional program for preventing bullying in the workplace or organizational policy for nurses. In addition, a more in-depth, repeated study is suggested on the relationship between workplace bullying experiences and workplace bullying responses according to individual countermeasures to workplace bullying.

## Figures and Tables

**Table 1:** General characteristics of participants (N = 113).

Characteristics	Categories	N (%)
Gender	Female	106 (93.8)
	Male	7 (6.2)
Marital status	Single	92 (81.4)
	Married	21 (18.6)
Education	College	51 (45.1)
	University	57 (50.4)
	Graduate school	5 (4.4)
Type of duty	Shift	106 (93.8)
	Day work	7 (6.2)
Age (years)	≤29	81 (71.7)
	30–39	29 (25.7)
	≥40	3 (2.7)
Religion	Yes	40 (35.4)
	No	73 (64.6)
Working unit	Medical unit	45 (39.8)
	Surgical unit	21 (18.6)
	Special unit	38 (33.6)
	Others	9 (8.0)
Total length of career (years)	<1	15 (13.3)
	1–5	54 (47.8)
	6–10	31 (27.4)
	>10	13 (11.5)

**Table 2:** Mean of workplace bullying experiences, responses, and ways of coping (N = 113).

Variables	Mean	SD	Min	Max
Workplace bullying	29.39	7.33	16.00	51.00
Verbal attacks and alienation	18.58	5.08	10.00	34.00
Improper work instructions	8.17	2.32	4.00	14.00
Physical threats	2.64	0.92	2.00	6.00
Consequences of workplace bullying	30.98	6.09	14.00	44.00
Physical and psychological withdrawal	19.75	4.10	8.00	30.00
Poor quality of patient care	4.57	1.26	2.00	8.00
Increasing distrust	6.66	1.99	3.00	12.00
Ways of Coping				
Problem-focused	22.19	2.51	15.00	28.00
Wishful thinking	12.60	1.76	5.00	17.00
Detachment	13.99	2.14	7.00	20.00
Seeking social support	18.21	2.28	12.00	24.00
Focusing on the positive	10.59	1.74	4.00	14.00
Tension reduction	7.42	1.57	3.00	12.00

**Table 3:** The difference of ways of coping according to general characteristics (N = 113).

Characteristics	Categories	Problem-Focused			Wishful Thinking			Detachment			Seeking Social Support			Focusing on the Positive			Tension Reduction		
		M	SD	t/F *p*	M	SD	t/F *p*	M	SD	t/F *p*	M	SD	t/F *p*	M	SD	t/F *p*	M	SD	t/F *p*
Gender	Female	22.22	2.45	−0.36 0.716	12.70	1.71	−2.31 0.023	14.02	2.12	−0.53 0.594	18.34	2.17	−2.36 0.202	10.61	1.68	−0.33 0.755	7.40	1.58	0.52 0.605
	Male	21.86	3.58		11.14	1.95		13.57	2.64		16.29	3.15		10.29	2.63		7.71	1.50	
Marital status	Single	22.16	2.59	−0.28 0.781	12.57	1.81	−0.46 0.646	14.13	2.24	1.46 0.148	18.24	2.31	0.26 0.795	10.68	1.78	1.18 0.242	7.62	1.54	2.99 0.003
	Married	22.33	2.20		12.76	1.58		13.38	1.50		18.10	2.19		10.19	1.54		6.52	1.40	
Education	College	22.39	2.15	1.09 0.339	12.71	1.63	0.23 0.795	14.33	2.05	1.24 0.292	18.10	2.41	0.74 0.479	10.45	1.53	0.73 0.483	7.24	1.49	0.82 0.441
	University	21.91	2.78		12.49	1.94		13.68	2.26		18.21	2.17		10.65	1.93		7.53	1.59	
	Graduate school	23.40	2.70		12.80	0.84		14.00	1.22		19.40	2.19		11.40	1.52		8.00	2.12	
Type of duty	Shift	22.25	2.41	0.99 0.325	12.63	1.75	0.71 0.479	14.01	2.15	0.35 0.726	18.26	2.28	0.94 0.349	10.65	1.70	1.39 0.169	7.50	1.54	2.26 0.026
	Day work	21.29	3.90		12.14	2.04		13.71	2.14		17.43	2.23		9.71	2.21		6.14	1.46	
Age (years)	≤29	21.96	2.72	1.88 0.158	12.58	1.82	0.02 0.979	13.95	2.12	0.34 0.710	18.14	2.43	1.82 0.167	10.57	1.86	0.28 0.759	7.53	1.57	1.64 0.199
	30–39	22.62	1.82		12.66	1.67		14.00	2.27		18.17	1.77		10.59	1.48		7.24	1.35	
	≥40	24.33	0.58		12.67	1.15		15.00	1.73		20.67	1.15		11.33	0.58		6.00	3.00	
Religion	Yes	21.93	2.31	−0.84 0.4010	13.18	1.65	2.63 0.010	14.28	2.36	1.04 0.299	18.63	1.86	1.43 0.155	10.60	1.91	0.03 0.975	7.33	1.59	−0.46 0.650
	No	22.34	2.62		12.29	1.75		13.84	2.01		17.99	2.46		10.59	1.66		7.47	1.56	
Working unit	Medical unit	21.80	2.63	1.84 0.144	12.87	1.53	1.99 0.127	13.64	1.88	1.14 0.338	18.20	2.58	0.20 0.894	10.27	1.94	1.91 0.132	7.31	1.68	0.26 0.856
	Surgical unit	22.05	2.56		12.05	2.22		14.19	2.54		18.52	2.29		10.38	1.32		7.52	1.36	
	Special unit	22.34	2.29		12.39	1.72		14.05	2.30		18.13	2.02		10.87	1.70		7.39	1.64	
	Others	23.89	2.37		13.44	1.51		15.00	1.41		17.89	1.90		11.56	1.42		7.78	1.30	
Total length of career (years)	<1	21.07	2.71	1.55 0.206	12.33	1.80	0.64 0.588	13.13	1.92	1.51 0.216	17.33	2.79	0.90 0.444	10.07	2.28	0.74 0.529	7.07	1.49	1.12 0.344
	1–5	22.15	2.83		12.50	1.94		13.89	2.29		18.41	2.42		10.61	1.80		7.61	1.56	
	6–10	22.74	1.67		12.97	1.52		14.52	2.06		18.23	1.76		10.87	1.36		7.48	1.59	
	>10	22.38	2.36		12.46	1.51		14.15	1.72		18.38	2.10		10.46	1.66		6.85	1.63	

**Table 4:** Correlation among workplace bullying, responses, and ways of coping (N = 113).

	1 r(*p*)	1.1 r(*p*)	1.2 r(*p*)	1.3 r(*p*)	2 r(*p*)	2.1 r(*p*)	2.2 r(*p*)	2.3 r(*p*)	3.1 r(*p*)	3.2 r(*p*)	3.3 r(*p*)	3.4 r(*p*)	3.5 r(*p*)	3.6 r(*p*)
1. Workplace bullying	1													
1.1. Verbal attacks and alienation	0.97 *** (<0.001)	1												
1.2 Improper work instructions	0.82 *** (<0.001)	0.67 *** (<0.001)	1											
1.3. Physical threats	0.57 *** (<0.001)	0.51 *** (<0.001)	0.29 ** (0.002)	1										
2. Consequences of workplace bullying	0.52 *** (<0.001)	0.48 *** (<0.001)	0.46 *** (<0.001)	0.30 ** (0.001)	1									
2.1. Physical and psychological withdrawal	0.29 ** (0.002)	0.26 ** (0.005)	0.30 ** (0.001)	0.14 (0.133)	0.92 *** (<0.001)	1								
2.2. Poor quality of patient care	0.60 *** (<0.001)	0.61 *** (<.001)	0.39 *** (<0.001)	0.44 *** (<0.001)	0.64 *** (<0.001)	0.41 *** (<0.001)	1							
2.3. Increasing distrust	0.59 *** (<0.001)	0.54 *** (<0.001)	0.55 *** (<0.001)	0.34 *** (<0.001)	0.76 *** (<0.001)	0.49 *** (<0.001)	0.50 *** (<0.001)	1						
3.1. Problem-focused	−0.23 * (0.013)	−0.24 * (0.011)	−0.14 (0.143)	−0.19 * (0.044)	−0.16 (0.086)	−0.10 (0.309)	−0.19 * (0.047)	−0.18 (0.057)	1					
3.2. Wishful thinking	−0.02 (0.859)	−0.01 (0.952)	0.01 (0.953)	−0.12 (0.213)	0.13 (0.184)	0.12 (0.221)	0.05 (0.568)	0.11 (0.238)	0.32 ** (0.001)	1				
3.3. Detachment	0.13 (0.186)	0.10 (0.280)	0.10 (0.305)	0.19 * (0.044)	0.15 (0.105)	0.08 (0.411)	0.12 (0.214)	0.23 * (0.013)	0.18 (0.064)	0.47 *** (<0.001)	1			
3.4. Seeking social support	0.01 (0.984)	−0.05 (0.620)	0.16 (0.101)	−0.12 (0.218)	−0.06 (0.513)	−0.06 (0.533)	−0.13 (0.162)	0.02 (0.867)	0.51 *** (<0.001)	0.30 ** (0.001)	0.18 (0.054)	1		
3.5. Focusing on the positive	−0.23 * (0.016)	−0.25 ** (0.008)	−0.10 (0.271)	−0.18 (0.052)	−0.27 ** (0.004)	−0.20 * (0.031)	−0.25 ** (0.008)	−0.24 ** (0.009)	0.56 *** (<0.001)	0.20 * (0.031)	0.25 ** (0.008)	0.36 *** (<0.001)	1	
3.6. Tension reduction	0.07 (0.479)	0.07 (0.497)	0.06 (0.517)	0.03 (0.791)	−0.06 (0.541)	−0.08 (0.427)	0.11 (0.245)	−0.09 (0.332)	0.21 * (0.027)	0.04 (0.665)	0.15 (0.126)	0.22 * (0.018)	0.36 *** (<0.001)	1

* *p* < 0.05; ** *p* < 0.01; *** *p* < 0.001.
